# Prevalence of Pre-Analytical Errors in Clinical Chemistry Diagnostic Labs in Sulaimani City of Iraqi Kurdistan

**DOI:** 10.1371/journal.pone.0170211

**Published:** 2017-01-20

**Authors:** Dereen Najat

**Affiliations:** Department of Chemistry, College of Science, University of Sulaimani, Sulaimani, Iraq; Gentofte Hospital, DENMARK

## Abstract

**Background:**

Laboratory testing is roughly divided into three phases: a pre-analytical phase, an analytical phase and a post-analytical phase. Most analytical errors have been attributed to the analytical phase. However, recent studies have shown that up to 70% of analytical errors reflect the pre-analytical phase. The pre-analytical phase comprises all processes from the time a laboratory request is made by a physician until the specimen is analyzed at the lab. Generally, the pre-analytical phase includes patient preparation, specimen transportation, specimen collection and storage. In the present study, we report the first comprehensive assessment of the frequency and types of pre-analytical errors at the Sulaimani diagnostic labs in Iraqi Kurdistan.

**Materials and Methods:**

Over 2 months, 5500 venous blood samples were observed in 10 public diagnostic labs of Sulaimani City. The percentages of rejected samples and types of sample inappropriateness were evaluated. The percentage of each of the following pre-analytical errors were recorded: delay in sample transportation, clotted samples, expired reagents, hemolyzed samples, samples not on ice, incorrect sample identification, insufficient sample, tube broken in centrifuge, request procedure errors, sample mix-ups, communication conflicts, misinterpreted orders, lipemic samples, contaminated samples and missed physician’s request orders. The difference between the relative frequencies of errors observed in the hospitals considered was tested using a proportional Z test. In particular, the survey aimed to discover whether analytical errors were recorded and examine the types of platforms used in the selected diagnostic labs.

**Results:**

The analysis showed a high prevalence of improper sample handling during the pre-analytical phase. In appropriate samples, the percentage error was as high as 39%. The major reasons for rejection were hemolyzed samples (9%), incorrect sample identification (8%) and clotted samples (6%). Most quality control schemes at Sulaimani hospitals focus only on the analytical phase, and none of the pre-analytical errors were recorded. Interestingly, none of the labs were internationally accredited; therefore, corrective actions are needed at these hospitals to ensure better health outcomes. Internal and External Quality Assessment Schemes (EQAS) for the pre-analytical phase at Sulaimani clinical laboratories should be implemented at public hospitals. Furthermore, lab personnel, particularly phlebotomists, need continuous training on the importance of sample quality to obtain accurate test results.

## Introduction

Accurate laboratory results are vital for patient safety and improving the medical diagnosis of patients, and many studies have shown that 70% of medical diagnostic decisions depend on the accuracy of laboratory tests. Despite advanced automation in diagnostic labs, there are still considerable error rates at clinical diagnostic labs [1].

**Fig 1:**
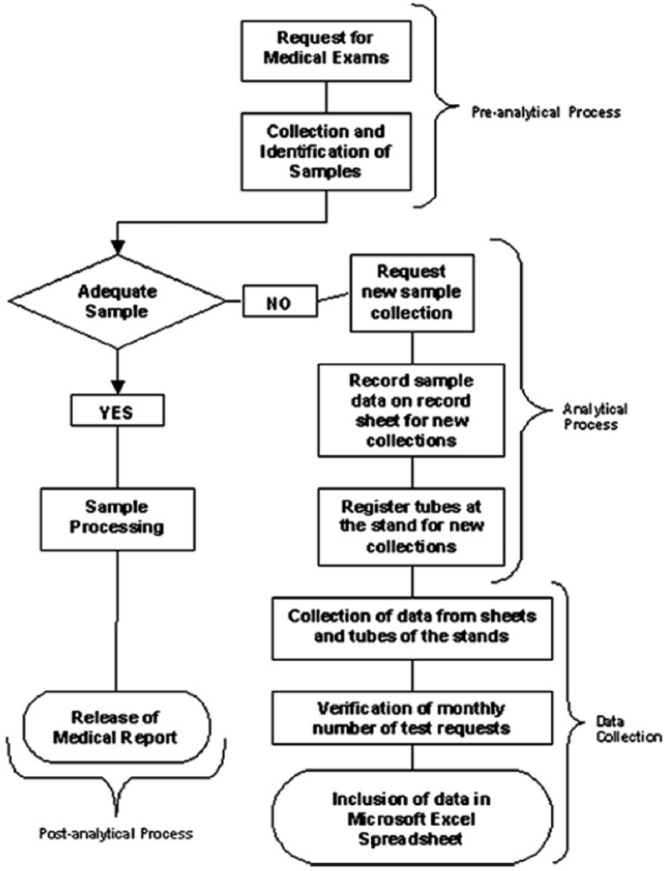
Flowchart illustrating the data collection process [3].

In clinical diagnostic laboratories, the total testing process includes every step from the test request to the receipt of results (Fig 1). The lab testing process generally comprises three phases. First is the pre-analytical phase, which according to the International Organization for Standardization (ISO) 15189:2012 standard for laboratory accreditation, encompasses all the steps from test request, sample collection, transport and registration of the sample up to the start of specimen analysis. Second is the analytical phase, which involves the analysis of the analytes and technical validation of the results. Third is the post-analytical phase, which includes the interpretation of the results, approval from the lab manager and reporting to the clinician [2]. Laboratory errors might occur at any of these three phases, and errors are not exclusive to the analytical phase. Errors lead to an increased demand of resources, inappropriate clinical decisions, delayed diagnoses and longer hospital stays [2].

Extra-analytical phases (pre- and post-analytical) have been recognized as a large source of laboratory errors, particularly the pre-analytical phase. Interestingly, a majority of diagnostic lab errors are either pre-analytical (46–68%) or post-analytical (18–47%). Indeed, only 7–13% of errors actually occur during the analytical phase [2]. Notably, the pre-analytical phase is the most crucial and hardest to regulate and monitor because of the involvement of too many professionals, such as physicians, specialists of laboratory medicine, nurses, laboratory technicians and phlebotomists [2].

Unlike the analytical phase, the extra-analytical phases are seldom subject to quality control schemes. Interestingly, the International Federation of Clinical Chemistry and Laboratory Medicine Working Group for Laboratory Errors and Patient Safety (IFCC-WG-LEPS) organized a series of quality markers on the pre-analytical phase to highlight pre-analytical phase errors [4]. The most common extra-analytical errors include inappropriateness of test order, patient identification error, timing errors in sampling and preparation, hemolytic and lipemic blood samples, inappropriate transport, and inadequate and inappropriate sample collection tubes [5] (Table 1).

**Table 1:** Types and description of most common pre-analytical errors [5].

Pre-analytical errors	Description
Hemolyzed sample	Presence of pink to red tinge in serum plasma
Insufficient sample	Serum obtained not enough for requested tests
Incorrect sample tube	Most samples received should not be in anticoagulated tubes
Sample not on ice	Samples for arterial blood gases analysis not transported on ice
Incorrect sample identification	Mismatch between name on sample and request form
Tube broken in centrifuge	The use of different tube sizes for sample collection
Delay in sample transportation	Samples were not sent to the laboratory on time
Expired reagents	Some reagents expired before use
Sample mix-ups	Samples intended for other laboratories were sent to the biochemistry laboratory

The purpose of the present study was to analyze the pre-analytical phase at selected diagnostic labs under Sulaimani governance to raise awareness of the importance of quality controls for the extra-analytical phases and implement international external quality assurance (EQA) at public hospitals. To this end, we analyzed the rate of and reasons for the rejected samples and measured the types and frequencies of pre-analytical errors. We also conducted a survey to study the various aspects of the pre-analytical and analytical phases at the laboratories.

## Materials and Methods

Over 2 months, 5500 venous blood samples were collected from 10 public diagnostic labs in Sulaimani City (550 samples were collected from each hospital). We documented the occurrence of pre-analytical phase errors from February to April 2016.

Each sample was followed from the time of blood withdrawal to testing equipment.

Each step of laboratory processing was recorded, including standard operating procedures for phlebotomy techniques, patient preparation, sample handling, instrument handling and maintenance.

Post analytical and analytical phase errors, such as broken probes, and faulty rotors, pumps and feeder systems, etc. were monitored to ensure that these errors did not occur during the present study.

Fully automated analyzers were used to analyze the samples. Equipment inbuilt with calibration traceability and internal quality controls (QC) were occasionally observed, and weekly calibrations were maintained. Any analytes observed out of range were subsequently recalibrated.

The percentage of each of the following pre-analytical errors were recorded: delay in sample transportation, clotted samples, expired reagents, hemolyzed samples, samples not on ice, incorrect sample identification, insufficient sample, tube broken in centrifuge, request procedure error, sample mix-ups, data communication conflict, order misinterpreted, lipemic samples, sample contaminated and physician’s request order missed. Percentage calculations were obtained by the ‘number of rejected samples’/ ‘total number of samples’ formula.

### The path of the samples

The samples were followed from the moment of blood withdrawal to vaccutainer transportation, centrifugation of the vaccutainers, waiting time and the time of the analysis.

The blood withdrawal procedure at the public hospitals is as follows:

Patients brought the requested tests on a piece of paper (from the hospital); the majority of blood draws were performed while the patient was sitting on a regular chair; however, during rush hours, some of the patients were standing during blood withdrawal. The blood was transferred to vaccutainers, and the phlebotomist inverted the vaccutainers 2–3 times. After the samples were labeled manually for name, age, gender and type of test, the samples were put on separate rack for each unit of laboratory tests. The samples were collected between 9–11 am.

After reaching the lab, the samples were centrifuged immediately and recorded in a special logbook.

Researchers recorded any inappropriateness. The specimens were allowed to clot, centrifuged at a speed of 3000 relative centrifugal force (RCF) for two minutes and then delivered to the analyzers.

### Survey design

Phlebotomist and laboratory personnel were interviewed and asked to fill out a survey (see Appendix 1) to assess their knowledge on pre-analytical errors. The survey consisted of 11 questions designed by Cornes *et al*. 2015. The survey was modified according to local lab requirements. The main aim of the survey was to evaluate the pre-analytical error records at our selected hospitals. The survey also questioned the type of platforms and systems used in the labs and whether analytical errors were recorded or not. Furthermore, the survey aimed to evaluate the attitude and knowledge of the selected labs regarding quality control programs and whether the labs were willing to participate in external quality assessment (EQA) schemes. The survey answers were manually recorded.

### Statistical analysis

All data were analyzed using SPSS software SPSS (Statistical Package for the Social Science; SPSS Inc., Chicago, IL, USA) version 21 for Microsoft Windows.

Data were collected and observed carefully from ten different public hospitals in Sulaimani city. The sums of errors were calculated. Their relative frequencies compared to the total specimens were also calculated and presented as a percentage. The difference between relative frequencies of errors observed in the hospitals considered was tested by a proportional Z test. P≤0.05 was considered statistically different.

#### Ethics statement

This study was performed according to the guidelines stated in the Declaration of Helsinki, and all procedures involving human subjects were approved through the ethical committee of Sulaimani University-Iraq. The data were anonymously analyzed. All the participants were notified of the goals of the study, and written consent was obtained (Appendix 2). There was no cost to the participants for the biochemical tests. All relevant forms are attached in Appendix 2.

## Results

During a two-month period, 5500 venous blood samples were analyzed in this study for pre-analytical errors. Each sample was followed strictly from the start of the blood test order by the clinician to the final reporting of the test results.

**Fig 2:**
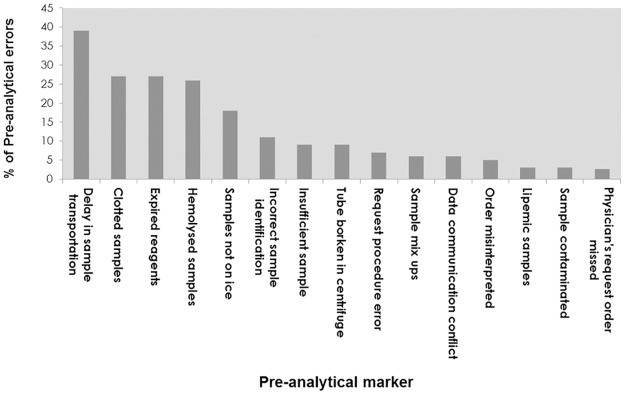
Frequencies and types of pre-analytical errors. A total of 15 types of pre-analytical errors were recorded at 10 different diagnostic labs in Sulaimani City. The pre-analytical errors are shown from the highest to the lowest frequency. The Fig 2 data is available as S1 Data.

**Fig 3:**
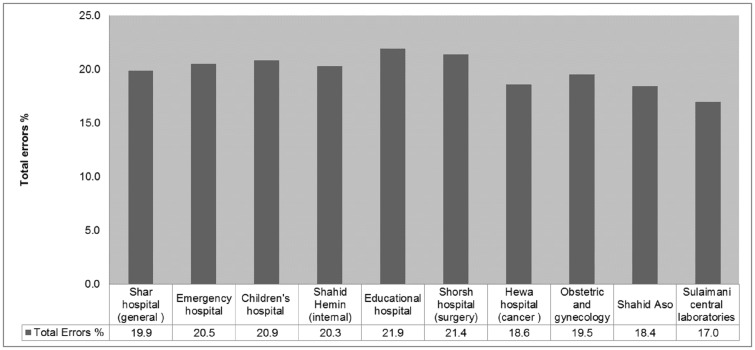
Pre-analytical error comparison at selected Sulaimani City hospitals. The difference between the relative frequencies of errors observed in hospitals was tested using a proportional Z test. The Central lab had the lowest error rate (17%), while the Shorsh hospital had the highest error rate (21.9%). There were no significant differences between the frequencies of the pre-analytical errors between the ten different hospitals, P = 0.231. The Fig 3 data is available as S3 Data.

The types and frequency of pre-analytical errors investigated in this study are shown in Fig 2. Our study showed a high prevalence of pre-analytical errors at selected Sulaimani diagnostic labs and that there was no significant difference between the ten hospitals in the frequency and types of pre-analytical errors (Fig 3). Delay in sample transportation, expired reagents, and hemolyzed and clotted samples were the most common types of errors, presenting 39%, 27%, 26% and 26%, respectively. Missing physician’s request orders was the least occurring error at Sulaimani labs (2.7%).

The types and most frequent errors and rejection rates are shown Table 2. The rejection rates for the errors were noticeably low, indicating the heavy usage of inappropriate samples at Sulaimani diagnostic labs. The main reasons for rejection Included hemolysis (9%), tubes broken in centrifuges (8%), expired reagents (7%) and inappropriate clotted samples (6%).

**Table 2:** Rejection rates and frequencies of pre-analytical errors at selected Sulaimani hospitals. The Table 2 data is available as S2 Data.

Pre-analytical errors	Frequency (%)	Rejection rate (%)
Delay in sample transportation	39	3
Clotted samples	27	6
Expired reagents	27	7
Hemolyzed samples	26	9
Samples not on ice	1	5
Incorrect sample identification	11	8
Insufficient sample	9	3
Tubes broken in centrifuge	9	8
Request procedure errors	7	2
Sample mix ups	6	3
Data communication conflict	6	3
Order misinterpreted	5	1
Lipemic samples	3	1
Contaminated samples	3	1
Missing physician’s request order	2.7	2

The survey responses (Appendix 1) provided the following results (n = 30):

Questions 1 and 2 examined how requests are counted at the lab, and the survey showed that requests were manually recorded.
Question 3 revealed that a majority (91%) of labs at Sulaimani hospitals use Roche instrumentation for hormone and vitamin measurements (models Cobas E 411, Cobas C 311 and Cobas 6000 analyzer series), and 7 of 10 labs used various spectrophotometers from the Biolabo Company. Manual spectrophotometers, such as the PD 303 spectrophotometer (APEL Co.) were also used.
Question 4 examined whether the labs used automated serum indices for hemolysis, icterus and lipemia (HIL), and the survey showed that none of the labs used an automated system to measure HIL indices.
Questions 5 and 6 examined whether laboratories recorded pre-analytical errors, and if recorded, were electronic platforms or manual recording used. The survey showed that none of the participating laboratories recorded pre-analytical errors.
Question 7 revealed that 52% of the participants were interested in working with international external quality assurance EQA programs, which compare local lab results with an unknown sample obtained from an internationally accredited lab.
Question 8 examined the participant’s interests in quality assurance indicators for the pre-analytical phase, and 63% of the participants were interested in guidance on the quality assurance indicators, while 52% of the participants responded to obtain specific quality assurance tests on laboratory information management systems (LIMS).
Questions 9–11 characterized educational program types to reduce pre-analytical phase errors, and 10% of the participants showed interest in centralized locations for such programs, while 52% of the participants favored local training at the work place. Only 7% of the participants showed interest in e-learning programs.

Table 3 summarizes the lab quality survey. The results obtained from the surveys answered by lab personnel at ten different hospitals. The survey investigated the level of quality control programs at the selected labs to gauge the skillset and attitude of the lab personnel.

**Table 3:** Summary of the lab quality survey.

No.	Survey Question results
1	The request sheets were manually recorded
2	None of the participating labs recorded pre-analytical errors
3	None of the selected labs used automated systems to assess HIL indices
4	63% of the participants showed interests in guidance on quality assurance indicators
5	52% of the participants were interested in international EQA programs
6	10% of the participants were interested in centralized locations for quality control programs
7	52% of the participants favored training at the work place, while only 7% of the participants showed interests in e-learning programs
8	91% of the selected labs used Roche instrumentation (models Cobas E 411, Cobas C 311 and Cobas 6000 analyzer series), a Biolabo spectrophotometer, and manual spectrophotometers, such as a PD 303 spectrophotometer (APEL Co.)

## Discussion

Pre-analytical phase errors have been the focus of research in past decades. Previous studies have focused on the analytical phase of diagnostic tests, and many quality control programs were initiated at diagnostic labs to monitor analytical phase errors. However, post- and pre-analytical errors were neglected worldwide, and currently many studies are focusing on the importance of the pre-analytical phase to obtain accurate lab results. An American pathologist program conducted a study enrolling 660 laboratories and showed that order error rate from outpatient centers was 4.8% [6]. The College of American Pathologists, including 120 labs, concluded that misidentification is a common laboratory error [7]. Interestingly, a Danish study on laboratory errors showed that 81% of lab errors were pre-analytical, while only 10% of lab errors were analytical. Moreover, 82.6% human errors and 4.3% technical errors were observed [7].

In the present study, we conducted the first examination of the types and frequencies of pre-analytical errors of laboratories in Sulaimani City, Kurdish region of Iraq. The results were consistent with those of previous studies [8–9] showing a high prevalence of pre-analytical errors. The majority of pre-analytical errors at selected Sulaimani hospitals included delays in transport, hemolysis, sample clotting and expired reagents. Interestingly, delayed specimen transport showed an alarming trend at selected hospitals, and many corrective actions are needed to minimize this type of error. Some of the labs at Sulaimani hospitals were located further from blood withdrawal rooms; the samples frequently underwent various temperature fluctuations before reaching the lab for analysis. Some of the samples were transported from the blood withdrawal room, which is not properly air-conditioned, and ambient temperatures typically range from 30–45°C from May-October. Blood withdrawal rooms should be located near diagnostic labs to minimize the effect of temperature changes, and specialized containers should be used to transport blood samples. Notably, untrained hospital staff (uneducated or have less than high school degrees) were transporting the samples. Studies showed that staff and trained phlebotomists make 2–4 times fewer pre-analytical errors than non-phlebotomists and lab staff [10]. A separate study showed that general practitioners and clinical hospital wards made about half of the pre-analytical errors [7].

Temperature fluctuations resulting from transport delay is a serious pre-analytical error, and many medical staffs are unaware of the instability of temperature-dependent analytes. Examples of temperature-sensitive diagnostic analytes include red and white blood cells, high-density-lipoprotein cholesterol, glucose, creatinine, total cholesterol, total testosterone, free testosterone, alkaline phosphatase, total bilirubin, thiobarbituric acid-reactive substances [11], glucose and potassium [12].

These results were similar to many other studies [6, 7, 13] showing a higher rate of hemolytic samples. However, the frequency of hemolytic samples might be over- or underestimated primarily because there are many other causes of high-rate *in-vitro* hemolysis, such as using serum rather than plasma, using a syringe to fill vaccutainers instead of a vacuum system, and personal errors arising from staff collecting blood. In addition, some types of illnesses might cause *in-vivo* hemolysis, contributing to the high incidence of hemolytic samples in the present study [13–14].

More importantly, many phlebotomists and lab staff should be reminded that hemolysis is one of the most common causes of pre-analytical errors, causing considerable harm to the accuracy of analytical tests. Analytes, such as alanine aminotransferase (ALT), aspartate aminotransferase (AST), creatinine, and creatine kinase (CK), are typically overestimated when hemolytic samples are used for analysis, while other analytes, such as albumin, alkaline phosphatase (ALP), chloride, g-glutamyltransferase (GGT), glucose and sodium, were reduced when hemolytic samples were used [15].

Expired reagents were used at selected hospitals, and clinicians should always be aware of this problem. Despite the high rate of the pre-analytical errors, the rejection rates of inappropriate samples were low, suggesting that a large number of inappropriate samples were sent for analysis, which in turn caused unnecessary test errors leading to incorrect clinical diagnoses and, on many occasions, unnecessary repetitions of many lab tests.

Our questionnaire revealed that most of the phlebotomists were not interested in enrolling in training and education programs and were reluctant to answer the questionnaire, reflecting inadequate English language skills. Therefore, we translated the questionnaire to encourage the participation of phlebotomists, but the response rate remained low.

Approximately 65% of lab personnel were also not interested in quality improvement programs or monitoring pre-analytical errors but were willing to participate in educational workshops concerning the pre-analytical phase.

Interestingly, the questionnaire revealed that lab personnel depend on the naked eye to detect hemolysis, icteric and lipemic samples, and spectrophotometric methods increased the detection rates of HILS; therefore, it is necessary to use spectrophotometric methods to detect HILS [13].

The present study has some limitations. For example, this study might be biased because the researcher subjectively evaluated HIL through visual inspection; instead, the use of automated spectrophotometric methods to accurately determine HIL, in which a quantitatively determined threshold is used to reject samples, is preferred.

Another limitation of this study is that some of the lab personnel were reluctant to answer the survey questions; therefore, these results were based on a small number of participants (n = 30), and obviously a larger number of participants are needed to draw firm conclusions from the survey.

In addition, this study was limited to a two-month observation in clinical chemistry labs at ten public hospitals. In the future, we plan to include a larger number of clinical chemistry labs at both public and private diagnostic labs in the city. Furthermore, investigating all diagnostic lab branches (hematology, virology and microbiology labs) at public hospitals is necessary to evaluate the types and trends of pre–analytical errors at other diagnostic labs.

## Conclusion

All ten diagnostic labs lacked vigorous quality control programs, and there is an urgent need to improve the quality control schemes at the selected hospitals; although internal quality controls have been used at these hospitals, the detected errors are primarily specific for the analytical phase, and the labs lack external quality control schemes. Notably, all neighboring countries (Turkey, Iran, Jordan, Saudi Arabia, Lebanon) are internationally accredited for all analytical phases. It is crucial to implement international external quality at these labs, and the College of American Pathologists (CAP) should monitor these labs. The CAP offers international services and extensive quality control assurance programs, assessments and many continuing educational programs. Thus, our government should make the extra effort to ensure equal quality at public hospitals.

## Appendix 1

### Quality control assessment survey modified from Cornes *et al*. 2015

1. How do you count requests? a. Each sample has a separate accession number b. Each request has a separate accession number
2. How do you record errors? a. Manual reporting b. LIMS based data collection
3. What analytical platform do you use? a. Roche b. Abbott c. Siemens d. Vitros e. Beckman f. Other? Please state
4. Do you use automated hemolysis, icterous, and lipemic (HIL) indices? a. Yes b. No
5. Do you currently routinely monitor any pre-analytical markers, such as hemolysis, non-received samples, insufficient samples, booking errors, etc.? a. Yes b. No
6. If you already monitor pre-analytical markers, what do you measure (check all that apply)? a. Illegible requests b. Percentage of samples where tests were not requested first time (add-ons)
7. Would you enroll in an EQA scheme to compare pre-analytical error rates with other institutions? a. Yes b. No
8. Would you be interested in any guidance documents on the best approach to collect data to ensure standardization? a. Yes, generic guidance b. Yes, guidance specific to LIMS systems c. No c. Percentage of samples not received d. Percentage of inappropriate samples received e. Percentage of Hemolyzed samples f. Percentage of lipemic samples g. Percentage of samples clotted h. Percentage of insufficient samples i. Percentage of samples received damaged j. Percentage of samples mislabeled k. Percentage of requests with missing samples l. Overall sample rejection rates m. Intralaboratory errors n. Booking errors o. Percentage of samples with delayed receipt (LOC) p. Other. Please state.
9. Would you be interested in a central location for guidance for the best pre-analytical practices? a. Yes b. No
10. Would you be interested in attending any day meetings to share initiatives? a. Yes b. No
11. Would you be interested in e-learning programs on pre-analytical monitoring and best practices? No

## Appendix 2

### A- Ethics approval was obtained from the Sulaimani University review board

A-Consent letter

Participant Code: ___

Title of Research: Prevalence of pre-analytical errors in clinical chemistry diagnostic labs in Sulaimani City of Iraqi Kurdistan

I agree to enroll in the study; I have read the letter of information, and the nature of the study has been explained to me. All questions I have regarding my participation in this study have been answered to my satisfaction. _______________________

Participant's Printed Name

_______________________ ______________________

Participant's Signature     Date

______________________________________

Printed Name of Person Obtaining Consent

_________________________________________ ____________________

Signature Name of Person Obtaining Consent       Date

_________________________________________ ____________________

### B-Socio-demographic, health and cultural factors questionnaire

Participant Code: ___

Title of Research Study: Prevalence of pre-analytical errors in clinical chemistry diagnostic labs in Sulaimani City of Iraqi Kurdistan

All information in this questionnaire is confidential and only reviewed by the members of the research team and ethics board.

Personal Information:

1-Date of birth (DOB) _________.
2-How long have you been in Sulaimani City?
  -Less than 1 year
  -2 years
  -3–5 years
  -5 years
3-Educational Level:
  -Primary
  -Secondary
  -Bachelor degree
  -Higher education
4-Occupation
5-Income:
6-Ethnic origin
  -Kurdish
  -Arabic
  -Other

## Supporting Information

S1 Data An excel file containing the data used to generate Fig 2, it contains collected number of samples with pre-analytical errors and percentage of pre-analytical errors. (XLSX)

S2 Data An excel file containing the data used to generate Table 2, it contains collected number of samples with pre-analytical errors and number of rejected samples. (XLSX)

S3 Data An excel file containing the data used to generate Fig 3, it contains collected number of samples with pre-analytical errors at each of the ten selected Sulaimani city hospitals. (XLSX)
